# Connexin 43 Affects Pulmonary Artery Reactivity via Changes in Nitric Oxide Production and Influences Proliferative and Migratory Responses in Mouse Pulmonary Artery Fibroblasts

**DOI:** 10.3390/ijms26031280

**Published:** 2025-02-01

**Authors:** Saad Wali, Abdmajid Hwej, David J. Welsh, Kathryn Wilson, Simon Kennedy, Yvonne Dempsie

**Affiliations:** 1 Pharmacology and Toxicology Department, College of Pharmacy, Umm Al-Qura University, Makkah 24342, Saudi Arabia; smwali@uqu.edu.sa; 2 School of Cardiovascular & Metabolic Health, College of Medical, Veterinary and Life Sciences, University of Glasgow, Glasgow G12 8TA, UK; abdmajid.hwej@glasgow.ac.uk; 3 Department of Biological and Biomedical Sciences, School of Health and Life Sciences, Glasgow Caledonian University, Glasgow G4 0BA, UK; david.welsh@gcu.ac.uk (D.J.W.); kathryn.wilson@gcu.ac.uk (K.W.); 4 School of Pharmacy, Univeristy of El-Mergib, Al-Khoms 11324, Libya

**Keywords:** connexin 43 (Cx43), gap junction, vascular reactivity, nitric oxide (NO), mouse pulmonary arterial fibroblasts (MPAFs), hypoxia, proliferation, migration

## Abstract

Pulmonary hypertension (PH) is a complex condition characterized by pulmonary artery constriction and vascular remodeling. Connexin 43 (Cx43), involved in cellular communication, may play a role in PH development. Cx43 heterozygous (Cx43^+/−^) mice show partial protection against hypoxia-induced pulmonary remodeling, with prior research highlighting its role in rat pulmonary artery fibroblast (PAF) proliferation and migration. However, inhibiting Cx43 may compromise nitric oxide (NO)-mediated vascular relaxation. This study evaluated the effects of Cx43 on mouse PAF (MPAF) proliferation, migration, NO-dependent and independent pulmonary vascular relaxation, and NO synthesis. Proliferation and migration were assessed in Cx43^+/−^ MPAFs under normoxic and hypoxic conditions. Vascular responses were analyzed in intra-lobar pulmonary artery rings with acetylcholine (ACh), SNAP, and U46619, while NO production was measured in lung tissue. Both genetic knockdown and pharmacological inhibition of Cx43 significantly reduced serum-induced proliferation but not migration under normoxia, while ^37,43^Gap27 inhibited hypoxia-induced proliferation and migration. The effects of genetic knockdown and pharmacological inhibition of Cx43 on vascular reactivity were also investigated. NO-dependent and independent relaxations and NO production were reduced in Cx43^+/−^ mice by ^37,43^Gap27. In conclusion, while Cx43 inhibition may protect against PAF proliferation and migration, it could also impair pulmonary vascular relaxation, at least in part through a reduction in NO signaling. Further studies are needed to fully understand the mechanisms by which Cx43 influences NO signaling.

## 1. Introduction

Pulmonary hypertension (PH) is a progressive and severe disorder characterized by elevated pulmonary artery pressure and resistance, leading to right heart failure and potential premature death. The pathophysiology of PH involves pulmonary vascular remodeling, which is characterized by the muscularization of pulmonary arteries, hypertrophy of the medial layer, and occlusive changes that increase resistance and vascular stiffness [1,2,3,4]. The impact of PH on patient quality of life is significant, and treatments are limited.

Connexins are crucial for enabling intercellular communication through gap junctions. Connexin 43 (Cx43) plays a significant role in vascular physiology and pathology [5,6]. Cx43 has extensive tissue expression in all cell types in the vasculature and regulates pulmonary vascular reactivity by modulating cell-to-cell communication and interacting with nitric oxide (NO) signaling pathways [7,8]. Pharmacological inhibition of Cx43 alters contractile responses to phenylephrine as well as vasodilators such as methacholine in pulmonary arteries, thereby affecting vascular tone and remodeling [8,9]. Additionally, Cx43 influences interactions between endothelial and smooth muscle cells in the pulmonary circulation [9,10].

Hypoxia causes constriction in the pulmonary vessels [11] and is also one of the most important factors driving cell proliferation and migration. Therefore, chronic hypoxia can lead to vasoconstriction, pulmonary vascular remodeling, and the development of pulmonary hypertension [12]. In fibroblasts, exposure to hypoxia can cause proliferation, migration, differentiation, upregulation of contractile and extracellular matrix proteins, and release of factors that directly affect smooth muscle cell tone and growth [13]. This has been observed across various species, including bovine, rats, mice, and humans [13,14,15,16], and is likely due to the release of mitogens [17,18,19]. Indeed, PAFs derived from PH patients and animal models of PH possess a pro-inflammatory, hyperproliferative, and apoptosis-resistant phenotype [20].

Based on recent reviews [21,22], Cx43 plays a complex role in cellular proliferation, displaying both pro- and anti-proliferative effects depending on the cell type and context. Cx43 facilitates the transfer of proliferative signals through gap junctions but can also contribute to contact inhibition, highlighting its dual regulatory capacity [23]. McNair et al. demonstrated that hypoxia-induced proliferation and migration in rat pulmonary arterial fibroblasts (PAFs) was reduced by inhibiting Cx43, emphasizing its importance in pulmonary vascular remodeling [10]. Moreover, it has been shown that hypoxic-induced pulmonary vascular remodeling was decreased in mice heterozygous for Cx43 (Cx43^+/−^ mice) [9]. Cx43 remains the most abundantly expressed connexin in human lung fibroblasts [24], and the gene encoding for Cx43, *GJA1*, is the most highly expressed in rat PAFs and PASMCs [10]. Therefore, the current study builds on these findings by investigating hypoxia-induced proliferation and migration in Cx43^+/−^ mice.

Connexins also have a role in vascular reactivity in the pulmonary circulation. For example, the contractile response to serotonin (5-HT) and phenylephrine was significantly decreased by pre-incubation with the gap junction blocker ^37,43^Gap27 in intra-lobar pulmonary arteries (IPAs) isolated from normoxic and chronic hypoxic (CH) rats [25]. In contrast, responses to some constrictors such as endothelin endothelin-1 (ET-1) were actually increased in IPAs derived from Cx43^+/−^ mice compared to wild-type (WT) mice [9,26]. Recently, Htet et al. found that the response to methacholine (MCh), which is an endothelial-dependent vasodilator, was significantly attenuated in IPAs derived from Cx43^+/−^ mice compared to WT mice and also in the presence of ^37,43^Gap27 [26]. Although effects on dilation may involve nitric oxide (NO), the effects of reduction of Cx43 expression on NO release in the pulmonary circulation have yet to be investigated.

Females are more likely to develop PAH compared to males [27], and this is reflected in a higher female-to-male ratio found in various registries, ranging from 1.4:1 in the UK/Ireland registry, 1.6:1 in the ‘European Comparative, Prospective Registry of Newly Initiated Therapies for Pulmonary Hypertension (COMPERA)’ registry, to as high as 4.1:1 in the ‘Registry to Evaluate Early and Long-term Disease Management in PAH (REVEAL)’ registry (reviewed in [28,29]). However, many pre-clinical studies continue to use single-sex groups of animals, which does not reflect the sex differences noted above. Consequently, this study compared IPAs from female and male WT and Cx43^+/−^ mice to study vasoreactivity and whole left lung NO production.

Although previous studies have measured the proliferation and migration of rat pulmonary artery fibroblasts (PAFs) and the influence of hypoxia and pharmacological inhibition of Cx43, here we have studied these processes in mouse PAFs (MPAFs) from Cx43^+/−^ mice. We hypothesize that Cx43 will influence MPAF proliferation and migration and affect vascular responses to vasodilators and constrictors. We speculate that effects on vascular relaxation will be caused by Cx43-induced changes in NO production.

## 2. Results

### 2.1. Effects of Cx43 on Proliferative Responses in MPAFs

**Figure 1:**
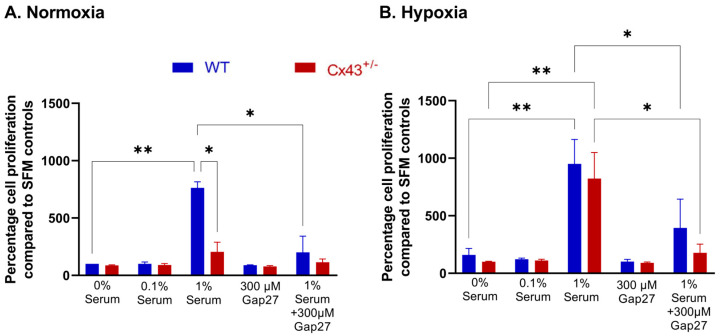
Effects of Cx43 on proliferation responses of MPAFs from WT and Cx43^+/−^ mice to serum under (**A**) normoxic and (**B**) hypoxic conditions (24 h). MPAFs from Cx43^+/−^ mice showed increased proliferation with 1% serum only under hypoxic conditions. However, treatment with ^37,43^Gap27 reduced 1% serum-induced proliferation in both normoxic and hypoxic conditions in WT MPAFs and under hypoxic conditions in Cx43^+/−^ MPAFs. Neither WT nor Cx43^+/−^ MPAFs exhibited proliferation in response to 0.1% serum in both normoxic and hypoxic conditions. Data are shown as mean ± S.E.M., and analysis was carried out by two-way ANOVA with a Tukey post hoc test. * *p* < 0.05, ** *p* < 0.01, n = 3.

MPAFs from WT mice proliferated in response to 1% serum under both normoxic and hypoxic conditions. MPAFs from Cx43^+/−^ mice were protected against serum-induced proliferation under normoxic but not hypoxic conditions. ^37,43^Gap27, a pharmacological inhibition of Cx37 and Cx43, reduced serum-induced proliferation of WT MPAFs under both normoxic and hypoxic conditions and Cx43^+/−^ MPAFs under hypoxic conditions. In the presence of 0.1% serum, MPAFs from WT and Cx43^+/−^ mice did not show any proliferative response under either normoxic or hypoxic conditions (Figure 1A,B).

### 2.2. Effects of Cx43 on Migratory Responses in MPAFs

**Figure 2:**
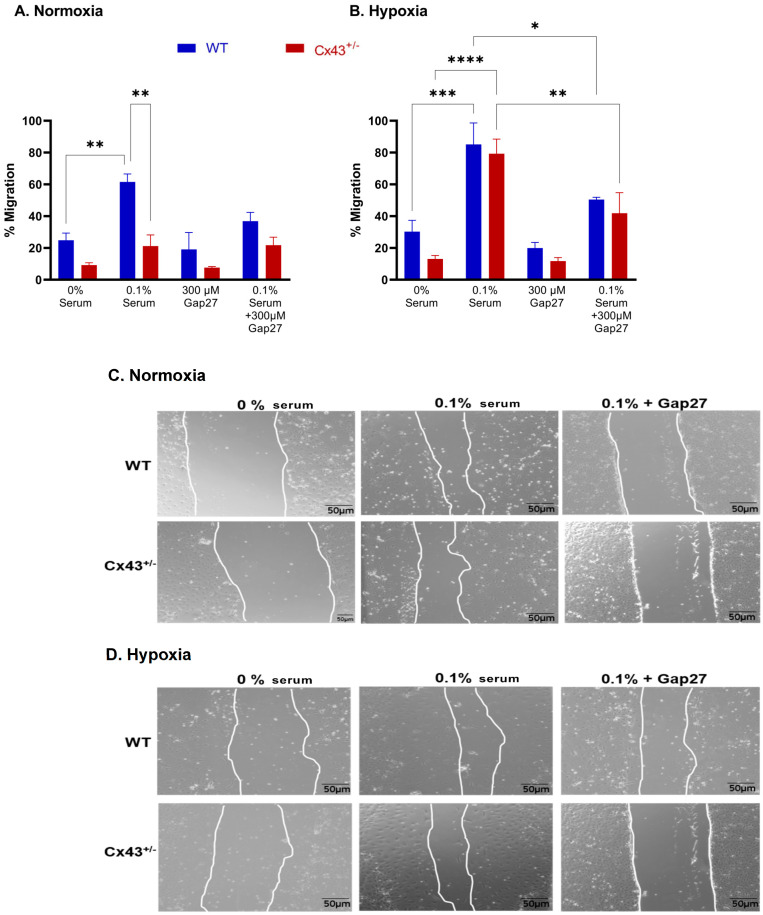
Effects of Cx43 on migration responses of MPAFs from WT and Cx43^+/−^ mice to serum under normoxic (**A**,**C**) and hypoxic (**B**,**D**) conditions (24 h). Under hypoxic conditions, MPAFs from Cx43^+/−^ mice showed increased migration with 0.1% serum, which was reduced by ^37,43^Gap27 in both WT and Cx43^+/−^ MPAFs. In normoxia, WT MPAFs exhibited a higher migratory response to 0.1% serum compared to Cx43^+/−^ MPAFs. The images in C and D depict the gap closure in scratch assays, indicative of cell migration. Data are shown as mean ± S.E.M., and analysis was carried out by two-way ANOVA with a Tukey post hoc test. * *p* < 0.05, ** *p* < 0.01, *** *p* < 0.001, **** *p* < 0.0001, n = 3.

MPAFs from WT mice had a migratory response to 0.1% serum under normoxic and hypoxic conditions. MPAFs from Cx43^+/−^ mice were protected against serum-induced migration under normoxic conditions but not hypoxic conditions. ^37,43^Gap27 significantly reduced migration of both WT and Cx43^+/−^ MPAFs under hypoxic, but not normoxic, conditions (Figure 2A,C). Scratch assay images (Figure 2C,D) illustrate the migratory patterns observed in MPAFs in response to varying serum concentrations.

### 2.3. Effect of Cx43 on Contractile Responses to U46619 in IPAs of Female and Male Mice

**Figure 3:**
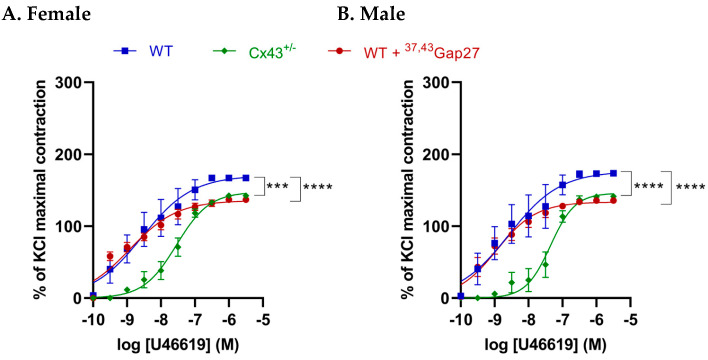
The contractile response to U46619 in IPAs of (**A**) female and (**B**) male mice. Both the maximum contraction (E_max_) and the logarithm of half-maximal effective concentration (LogEC_50_) to U46619 were significantly reduced in Cx43^+/−^ mice compared to WT mice. Additionally, the Cx43 blocker ^37,43^Gap27 markedly decreased U46619 responses in WT mice. No significant differences between sexes were found in either LogEC_50_ or E_max_. *** *p* < 0.001, **** *p* < 0.0001 compared to WT controls within same experimental group, n = 6 per group. Data are shown as mean ± S.E.M.

Concentration-response curves (CRCs) were constructed for the thromboxane A2 (TP) receptor agonist U46619 (0.1 nM–10 µM) in both female and male WT and Cx43^+/−^ mice. The maximal contractile response (E_max_) and the logarithm of half-maximal effective concentration (LogEC_50_) to U46619 were significantly decreased (*p* < 0.0001) in female and male Cx43^+/−^ mice compared to corresponding WT mice. In addition, pharmacological inhibition of Cx43 using ^37,43^Gap27 significantly reduced the contractile response to U46619 (*p* < 0.0001) in both female and male WT mice. No significant differences between sexes were found in either LogEC_50_ or E_max_ (Figure 3, Table 1).

### 2.4. Effect of Cx43 on Relaxation Responses to ACh in IPAs of Female and Male Mice

**Figure 4:**
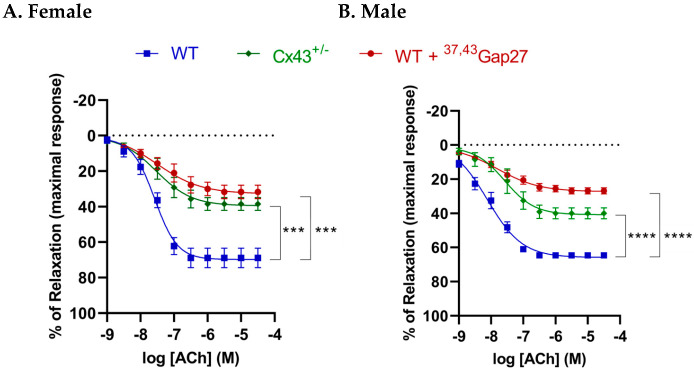
Endothelium-dependent relaxation to ACh in IPAs of (**A**) female and (**B**) male mice. A noticeable decrease in maximal relaxation responses in Cx43^+/−^ mice compared to wild-type controls across both sexes was observed. In addition, pre-incubation with the gap junction blocker ^37,43^Gap27 led to a reduction in ACh-induced relaxation in WT mice. However, no significant differences between sexes were found in either LogEC50 or R_max_. *** *p* < 0.001, **** *p* < 0.0001 compared to WT controls within same experimental group, n = 6 per group. Data are shown as mean ± S.E.M.

The maximal relaxation (R_max_) response induced by the endothelium-dependent dilator acetylcholine (Ach) was significantly decreased in both female (*p* < 0.001) and male (*p* < 0.0001) Cx43^+/−^ mice compared to corresponding WT mice. In addition, relaxation to ACh was also reduced by pre-incubation with the gap junction blocker ^37,43^Gap27 in both female (*p* < 0.001) and male (*p* < 0.0001) WT mice. However, no significant differences between sexes were found in either LogEC_50_ or R_max_ values (Figure 4, Table 2).

### 2.5. Effect of Cx43 on Relaxation Responses to SNAP in IPAs of Female and Male Mice

**Figure 5:**
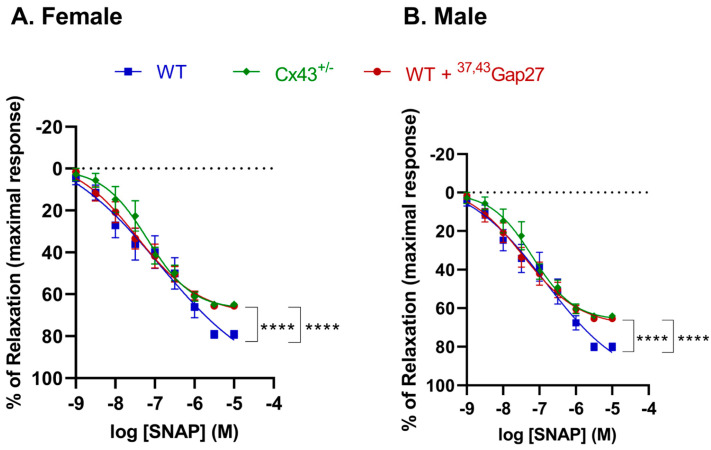
Relaxation produced by SNAP in IPAs of (**A**) female and (**B**) male mice. Reduced maximal relaxation (R_max_) was seen in female and male Cx43^+/−^ mice compared to WT controls. Pre-treatment with ^37,43^Gap27 also diminished relaxation responses in WT mice. However, no significant differences between sexes were found in either LogEC_50_ or R_max_. **** *p* < 0.0001 compared to WT controls within same experimental group, n = 6 per group. Data are shown as mean ± S.E.M.

ACh relaxes blood vessels by receptor-mediated generation of NO from endothelial cells. As there was a reduction in relaxation in response to ACh in the Cx43^+/−^ mice, we assessed whether relaxation to the endothelium-independent vasodilator, S-Nitroso-N-acetyl-DL-penicillamine (SNAP), was similarly affected. The maximal relaxation (R_max_) response to SNAP was significantly decreased (*p* < 0.0001) in Cx43^+/−^ IPAs compared to corresponding WT mice. Relaxation to SNAP was also reduced (*p* < 0.001) by pre-incubation with the gap junction blocker ^37,43^Gap27 in both male and female WT mice. However, no significant differences between sexes were found in either LogEC_50_ or R_max_ values (Figure 5, Table 3).

### 2.6. Investigating the Production of Nitric Oxide (NO) in the Whole Left Lung Lobe from Female and Male WT and Cx43^+/−^ Mice

**Figure 6:**
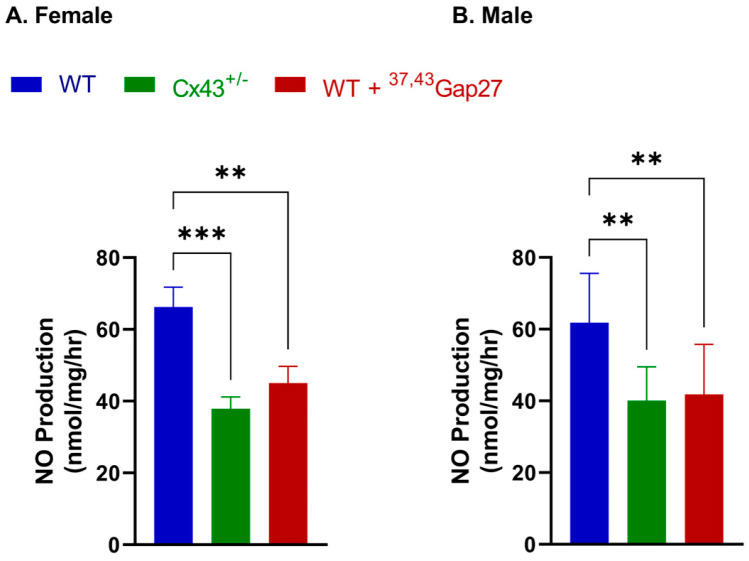
NO production by IPAs in whole left lung lobes. Panels (**A**,**B**) show NO production in female and male mice, respectively, revealing significant reductions between WT and Cx43^+/−^ mice. Addition of a Cx43 blocking peptide also significantly reduced NO production in both female and male WT left lung lobes. ** *p* < 0.01, *** *p* < 0.001 compared to WT within same experimental group. Data represent mean ± S.E.M. NO production was analyzed by two-way ANOVA with Tukey post hoc test, n = 7 per group.

Based on the reduction in relaxation observed in response to ACh in IPAs from Cx43^+/−^ mice, we decided to measure the basal NO formation by the whole left lung lobe using a Sievers NO analyzer. The production of NO was significantly decreased in the presence of ^37,43^Gap27, as well as in the Cx43^+/−^ mice compared to the WT mice, but no significant differences between sexes were found (Figure 6A,B).

## 3. Discussion

This study assessed Cx43′s regulation of MPAF proliferation and migration under normoxic and hypoxic conditions, and the role of Cx43 in vascular reactivity and its association with the NO signaling pathway. Findings indicate that both genetic and pharmacological modulation of Cx43 significantly affect MPAFs’ proliferation and migration, as well as arterial vasorelaxation and contraction. In addition, pharmacological inhibition or genetic reduction of Cx43 resulted in reduced NO generation within the lung. This research underscores Cx43′s crucial role in pulmonary vascular dynamics and the response to hypoxia.

MPAFs derived from WT mice had a proliferative and migratory response to serum under both normoxic and hypoxic conditions, which was inhibited by ^37,43^Gap27. MPAFs derived from Cx43^+/−^ mice did not proliferate or migrate under normoxic conditions but exhibited a similar proliferative and migratory response to MPAFs derived from wild-type mice under hypoxic conditions. Hypoxic-induced proliferative and migratory responses were inhibited by ^37,43^Gap27. A previous study has shown that hypoxic-induced proliferation and migration of rat PAFs were inhibited by both ^37,43^Gap27 and Cx43 siRNA [10]. Therefore, it may be that there are compensatory changes in Cx43^+/−^ mice in the expression of genes involved in hypoxic-induced proliferation and migration, allowing a full proliferative and migratory response in PAFs derived from these mice. Indeed, we have previously shown changes in the expression of the genes encoding Cx37, Cx40, Cx45, and pannexin-1 in Cx43^+/−^ mice [26].

In other areas of research, it has been shown that connexins, specifically Cx43, play a role in the migration of cancer cells. For example, a study conducted by Ogawa et al. demonstrated that suppression of Cx43 expression in rat hepatocellular carcinoma cells using Cx43-siRNA reduced invasion and migration capacity in vitro, as well as metastatic ability in-vivo, which suggests that Cx43 may be a target for drugs to prevent cancer metastasis in tumors that overexpress Cx43 [30]. In addition, Cx43 has been found to contribute to the increased malignancy and progression of prostate cancer by enhancing cell migration and invasion, specifically through elevated gene and protein expression levels in more metastatic prostate cancer cell lines [31]. Therefore, these studies have demonstrated that Cx43 plays an important role in cell migration, and there is a need to investigate the underlying mechanism.

The proliferation and migration of MPAFs from Cx43^+/−^ mice under hypoxic, but not normoxic, conditions can be attributed to several factors. For example, hypoxia triggers various signaling pathways and transcription factors, such as hypoxia-inducible factors that regulate cell survival and adaptation. It is possible that the lack of responsiveness in Cx43^+/−^ MPAFs to serum alone is because these MPAFs require the supplementary trigger of hypoxia [32]. Further research is needed to fully understand the underlying mechanisms behind the observed proliferative and migratory responses of MPAFs from Cx43^+/−^ mice under hypoxic conditions. In addition, the role of Cx43 in other pathological environments relevant to PH remains to be investigated.

This study also investigated the effects of both genetic knockdown and pharmacological inhibition of Cx43 on contractile responses to U46619 in IPAs of female and male mice. In both sexes, the contractile effects of U46619 were significantly reduced in the presence of ^37,43^Gap27 as well as in the Cx43^+/−^ mice compared to WT mice. These results are consistent with those obtained by Billaud et al., who found that the contraction to serotonin (5-HT) in IPAs from normoxic rats and the contraction to phenylephrine in IPAs from monocrotaline (MCT) and CH rats were substantially decreased after treatment with ^37,43^Gap27 [25]. More recently, using mouse pulmonary arteries under normoxic conditions, the contractile response and potency of phenylephrine were significantly attenuated in Cx43^+/−^ mice compared to WT mice [9]. The reduction in contraction to U46619 in pulmonary arteries of Cx43^+/−^ mice, despite a lower production of NO in the whole lung, raises an important point. Gap junctions and their subunit connexins might be involved in pulmonary vasoreactivity via their relationship with calcium (Ca^2+^). For example, calmodulin, which is the main receptor for Ca^2+^ ions in most tissues, was found to be associated with gap junctions and plays a direct role in chemical gate control of Cx32-containing gap junctions in frog oocytes [33]. In addition, two distinct amino acid sequences have been identified in Cx32 that are capable of binding calmodulin [34]. A study by Lurtz and Louis [35] showed that corresponding physiological concentrations of [Ca^2+^]_i_ in HeLa cells that were transfected with Cx43 were able to modify the permeability of Cx43 via the interaction between calmodulin and a cytoplasmic region of Cx43 in response to physiological concentrations of [Ca^2+^]_i_. It is well known that the concentration of intracellular calcium [Ca^2+^]_i_ is involved in modulating smooth muscle cell-membrane potential, and gap junctions are known to play an important role in controlling the function of adjacent smooth muscle cells in terms of both membrane potential and [Ca^2+^]_i_ [36,37]. Moreover, Srisakuldee et al. found that blocking Cx43 channels using ^37,43^Gap27 reduces [Ca^2+^]_i_ in the subsarcolemmal mitochondria of rat hearts [38]. Consequently, the Cx43^+/−^ mice used in this study may have reduced levels of calmodulin and intracellular Ca^2+^, resulting in decreased NO production and attenuated contraction in response to U46619. Additionally, compensatory mechanisms or the involvement of other vasoactive substances, such as increased EDHF or PGI_2_, may also counterbalance the effects of decreased NO. In addition, alterations in the expression or function of other connexins or gap junction proteins in Cx43^+/−^ mice could be influencing the observed reduction in contractile responses [26]. Hence, understanding these complexities will require further investigation, considering regional differences in vascular beds and specific cell types involved in the pulmonary arteries.

The present study also examined the effects of both genetic knockdown and pharmacological inhibition of Cx43 on relaxation responses to the endothelium-dependent vasodilator ACh in IPAs. The relaxation responses to ACh were significantly inhibited in Cx43^+/−^ mice and in the presence of ^37,43^Gap27 compared to WT mice. These results support the previous findings from Htet et al., who found that ^37,43^Gap27 reduced relaxation to the endothelium-dependent vasodilator methacholine (MCh) in IPAs of both female and male mice and in Cx43^+/−^ mice [26]. Our data indicate that Cx43 is likely involved in not only endothelium-dependent responses via effects on NO production but also in communication between the endothelium and vascular smooth muscle (VSMC) and how the VSMCs respond to vasoactive mediators and mitogens. This is supported by data shown in Figure 6, where there was a significant reduction in NO-mediated vasodilation by left lung lobes from Cx43^+/−^ mice and after treatment of lung lobes from WT mice with ^37,43^Gap27. More recently, Mondejar-Parreño et al. found that rat pulmonary arteries transfected with microRNA-1 (miR-1), which inhibits the gap junction protein alpha 1 (Gja1) gene encoding Cx43, showed a decreased relaxant response to Ach, which was associated with Cx43 downregulation [39]. Interestingly, miR-1 was the most highly upregulated miR in plasma from idiopathic pulmonary hypertension patients (8–12-fold increase) [40]. In addition, lungs from Sugen/hypoxic rats displayed a substantial upregulation of miR-1 [41]. Hence, the present study supports the previous literature that Cx43 is involved in vasorelaxation of the pulmonary vasculature and that blocking Cx43 channels or reducing the expression of Cx43 reduced relaxation responses.

The relaxation to an endothelium-independent dilator S-Nitroso-N-acetyl-DL-penicillamine (SNAP) was also reduced by both genetic knockdown and pharmacological inhibition of Cx43. Considering that Cx43 is expressed in both endothelial and smooth muscle cells, which is pivotal for cellular communication [42], these findings underscore the role of Cx43 in regulating endothelium-dependent and -independent relaxation pathways in pulmonary circulation. SNAP, which directly releases nitric oxide (NO) to mediate vascular relaxation without the need for endothelial interaction, might exhibit reduced effectiveness in Cx43-modified mice due to potential disruptions in NO handling or signaling at the smooth muscle level. This suggests that Cx43 may play an important role in modulating the bioavailability or efficacy of NO, warranting further investigation into how Cx43 alterations affect SNAP-induced vascular responses.

Due to the reductions in relaxation to ACh in Cx43^+/−^ mice, the production of basal NO was then measured in the whole left lung lobe. The production of NO was significantly attenuated by ^37,43^Gap27 as well as in the Cx43^+/−^ mice compared to the WT mice. Interestingly, Zhang et al. found that NO bioavailability was significantly reduced in PAH patients, and this could potentially contribute to the worsening of conditions through adverse effects of pulmonary blood flow [43]. It would be interesting if future studies assess whether NO production in response to agonists, such as ACh, is similarly affected in the Cx43^+/−^ mice and also investigate the link between Cx43 expression and function and NO production in pulmonary vessels more fully, especially since drugs augmenting NO-mediated vasodilation such as sildenafil are widely used to treat PAH patients.

The findings from the current study did not uncover any differences between sexes on (i) contractile responses to U46619, (ii) relaxation responses to ACh and SNAP, and (iii) NO production. It is important to mention that we did not investigate sex differences in the proliferation and migration of MPAFs. Interestingly, previous studies have uncovered some differences between female and male mice, albeit with different constrictor agents. For example, [26] observed notable sex differences in the vasoreactivity of IPAs. They found that ET-1 was more potent in IPAs from male Cx43^+/−^ mice compared to male WT mice, with no changes in the maximal responses. On the other hand, in female mice, contractile responses to ET-1 were no different [26]. However, both female and male Cx43^+/−^ mice had similar contractile responses to 5-HT when compared to WT controls. Therefore, these differences in contractile responses between sexes could be due to the agonists used, which supports the conclusions of [25] that the effects of ^37,43^Gap27 on contractile responses to phenylephrine, 5-HT, and ET-1 in IPAs varied based on the agonists used. Therefore, although no sex differences were found in the current study, it is still important to study differences between sexes in all pre-clinical and clinical research.

## 4. Materials and Methods

### 4.1. Ethical Statement

All animal procedures were carried out in accordance with Directive 2010/63/EU of the European Parliament. Ethical approval for the Cx43^+/−^ mouse studies was granted by the University of Strathclyde Animal Welfare and Ethics Committee (PPL no: PEE7AAC2C).

### 4.2. Materials

All primers for genotyping were purchased from Integrated DNA Technologies (IDT, Leuven, Belgium). All antibodies used in Western blot analysis were from Sigma Aldrich, Dorset, UK and Cell Signaling Technology, Danvers, MA, USA. All other chemicals and reagents were from ThermoFisher, Loughborough, UK or Sigma Aldrich, unless otherwise stated. S-Nitroso-N-acetylpenicillamine (SNAP) (BML-CN210-0020) was obtained from Enzo, Exeter, UK. ^37,43^Gap27 (A14866-25) was obtained from Adooq Bioscience, Irvine, CA, USA. Peptide controls for ^37,43^Gap27 have been carried out previously [44,45].

### 4.3. Animals

The Cx43 heterozygous (Cx43^+/−^) mice used in these studies were generated by homologous recombination in embryonic stem cells via insertion of a neomycin resistance gene (neo^r^) into exon 2 of the Cx43 (GJA1) gene (detailed in [46]). Homozygous (Cx43^−/−^) mice are not viable [46]. Wild-type (WT) and Cx43^+/−^ mice were bred in-house and housed under standard laboratory conditions. All mice had access to food and water ad libitum.

### 4.4. Genotyping

Mice were ear-notched for identification, and the ear notches were stored at −20 °C prior to genotyping. DNA was extracted, amplified, and analyzed using PCR to detect Cx43 (GJA1) and neomycin resistance (neo^r^) genes. Details of the DNA extraction, PCR conditions, primer sequences, and gel electrophoresis procedures have been described in detail in [26].

### 4.5. Primary Culture of MPAFs

Main and branch pulmonary arteries (diameter ~0.5–1.5 mm) were dissected free from the lung of female mice, cut longitudinally, and opened. A sterile razor blade was used to gently abrade and remove muscular tissue and the endothelial layer. To harvest enough fibroblasts, two pulmonary arteries were cut into 5 mm^2^ pieces and distributed over the base of a 25 cm^2^ culture flask containing 2 ml of Dulbecco’s modification of Eagle’s medium (DMEM) supplemented with 20% fetal bovine serum (FBS) plus 100 µg/mL primocin and 2 mM L-glutamine. The explants were incubated in a humidified atmosphere of 5% CO_2_ at 37 °C for three days before media were refreshed and tissue fragments were removed by aspiration. We have previously shown by staining for vimentin that this technique provides a pure culture of fibroblasts [12]. Cells were grown in 75 cm^2^ culture flasks containing 10 mL of DMEM supplemented with 20% FBS, 100 µg/mL primocin, and 2 mM L glutamine. Cells were sub-cultured into fresh growth medium once they reached ~80–90% confluency and used between passages 3 and 7. A humidified temperature-controlled incubator (Wolf Galaxy R, Nottingham, UK) was used as a hypoxic chamber. For hypoxic experiments, primary cultures of MPAFs were quiesced in serum-free media (SFM) for 24 h and then transferred to hypoxic conditions (5% O_2_, 5% CO_2_, and balance N_2_) for 24 h.

### 4.6. Proliferation Assay

An automated cell counter (Countless II, ThermoFisher, Loughborough, UK) was used to assess cell viability and proliferation. Cells were plated on a 24-well plate and grown to approximately 60% confluency, then quiesced for 24 h using SFM. SFM, 0.1% serum, and 1% serum were used. SFM served as the negative control, while 1% serum was used as the positive control. Cells were then treated with 300 µM of ^37,43^Gap27 and incubated in normoxic or hypoxic conditions for a further 24 h. After 24 h, the media was removed, and cells were washed twice with 500 µL of sterile phosphate-buffered saline (PBS). Cells were trypsinized (100 µL) for 3 mins and harvested with 500 µL of DMEM before being transferred into 1.5 ml centrifuge tubes and spun at 2600× *g* for 5 min at 4 °C. The cell pellet was then resuspended in 10 µL SFM, and 10 µL of 0.4% Trypan blue stain was added and loaded onto a glass slide and inserted into the Countless II cell counter to determine cell concentration per milliliter. The viable cell concentration was measured in triplicate samples. Percentage change in cell proliferation was calculated compared to normoxic SFM cells.

### 4.7. Migration Assay

A scratch was made using a sterile surgical blade on the bottom of each 6-well plate as a reference point before cells were cultured and left to grow until 100% confluency was reached. Cells were then quiesced for 24 h in SFM, an initial scratch was made in line with the reference scratch using a pipette tip, and the wells were photographed using Evos (ThermoFisher, Loughborough, UK). SFM was used as a negative control, while 0.1% serum was used as a positive control. This concentration of serum was insufficient to induce proliferation. In some wells, cells were treated overnight with 300 µM of ^37,43^Gap27. Cells were then incubated in normoxic or hypoxic conditions for a further 24 h. After 24 h, cells were photographed again for comparison. Scratch or migration assay experiments were performed in a minimum of three replicate plates and using cells isolated from three different animals. The extent of migration was made by measuring the percentage narrowing of the width of the scratch made in the cell layer in four different locations.

### 4.8. Tissue Preparation

Whole left lung lobe and intra-lobar pulmonary arteries (IPAs) were removed from 2- to 3-month-old male and female WT and Cx43^+/−^ littermate mice (C57BL/6 background). Mice were euthanized by intraperitoneal injection using pentobarbital sodium (Euthatal^®^) (60 mg/kg) plus lidocaine (4 mg/kg). The tissues were transferred into a petri dish containing cold physiological salt solution (PSS) (the composition of PSS was (mM): 119.0 NaCl, 4.7 KCl, 1.2 MgSO_4_, 24.9 NaHCO_3_, 1.2 KH_2_PO_4_, 11.1 Glucose, and 2.5 CaCl_2_).

### 4.9. Wire Myography Studies

Following the dissection and isolation of IPAs, the vessels were cut into 2 mm segments. Two wires of 2 cm in length and 40 μm in diameter were then carefully inserted into the lumen, and the artery was mounted on a wire myograph (Multi Myograph Model 610M, Danish Myo Technology, Denmark). Vessels were gassed (95% O_2_, 5% CO_2_), maintained at 37 °C in PSS, and allowed to equilibrate for 30 min. After equilibration, the vessels were normalized to reach a target pressure of 12–15 mmHg. LabChart™ v8 Pro software (ADInstruments, Chalgrove, UK) was used to record vessel tension. To check vessel viability, contraction to 3 additions of 60 mM of potassium chloride (KCl) was measured at the start of the experiments. Vessels were then pre-constricted using 30 nM of the thromboxane A2 (TP) receptor agonist U46619, and relaxation responses were carried out using cumulative additions of the endothelium-dependent vasodilator ACh (0.1 nM–30 µM) or the nitric oxide donor SNAP (0.1 nM–30 µM). In addition, CRCs to U46619 (0.1 nM–10 µM) were constructed in vessels at baseline tension. To check the effect of pharmacological inhibition of Cx43 on the function of IPAs, rings were pretreated for 30 mins with 100 µM of ^37,43^Gap27.

### 4.10. Nitric Oxide (NO) Assay

The whole left lobe of the lungs from female and male WT and Cx43^+/−^ mice were collected and weighed and then incubated for 30 min in 1 ml oxygenated physiological buffer solution at 37 °C and pH 7.4. The composition of the physiological buffer solution was the following (in mM): 119.0 NaCl, 4.7 KCl, 1.2 MgSO_4_, 24.9 NaHCO_3_, 1.2 KH_2_PO_4_, 11.1 Glucose, and 2.5 CaCl_2_. After 30 min, 100 μL of the conditioned media was collected, which contains substances released from the tissues into the media during the incubation period. Then, 400 μL of methanol (*v*/*v*) was added, and the mixture was centrifuged at 21,910× *g* at 4 °C for 20 min to precipitate unwanted proteins. The supernatant was then collected and kept at −80 °C and then assayed for NO content using a Sievers 280 NO analyzer (ThermoFisher, Loughborough, UK. The NO analyzer calculates the amount of NO produced by the tissues by calculating the amount of nitrite (NO_2_⁻), which was formed by the reaction between NO released from the tissues and the dissolved oxygen in the cell culture medium. Prior to each experiment, a standard curve of NO_2_⁻ was constructed from a standard solution of 100 mM sodium nitrite (NaNO_2_), and serial dilutions of 50 μM, 10 μM, 1 μM, and 100 nM were prepared and injected into the purge vessel using an Exmire microsyringe (ITO Corporation, Tokyo, Japan). The NO is produced in the purge vessel and is then detected by the NO analyzer via a reaction with oxygen (O_2_) to produce trioxygen or ozone (O_3_), which was detected by chemiluminescence. The chemiluminescence signal was converted to an electrical potential and displayed as millivolts (mV) by the NO analyzer. The amount of NO produced by duplicates of each NO_2_⁻ standard recorded by the analyzer was used to produce a calibration curve.

### 4.11. Statistical Analysis

Data were analyzed using GraphPad Prism 8.0 software. For cell culture and NO assay, comparisons between groups were made using two-way ANOVA followed by a Tukey post hoc test. For myography studies, changes in the logarithm of median effective concentration (LogEC_50_), relaxation responses (R_max_), and maximal contraction (E_max_) between different groups were analyzed using two-way ANOVA followed by Bonferroni’s post hoc test. Data are presented as mean ± standard error of the mean (S.E.M). A *p*-value of less than 0.05 was considered statistically significant, and the *n* symbol indicates the number of independent experiments.

## 5. Conclusions

In conclusion, we have found that Cx43 has a role in mediating vasoreactivity within the pulmonary circulation, with a close relationship to the nitric oxide (NO) signaling pathway. We have also established that Cx43 significantly influences the proliferation and migration of MPAFs under hypoxic conditions. These findings reinforce the notion of Cx43 as a pivotal factor in the cellular mechanisms leading to vascular remodeling in response to hypoxia; however, additional research is required to uncover the precise mechanisms through which Cx43 influences cellular behaviors and vasoreactivity.

## Figures and Tables

**Table 1:** The contractile effects of U46619 in IPAs from female and male WT and Cx43^+/−^ mice.

Agonist	Groups		LogEC_50_ (M)	E_max_ (%)	n
U46619	Female	WT Cx43^+/− ^ +^37,43^Gap27	−8.55 ± 0.21 −7.55 ± 0.08 *** −8.92 ± 0.09	170.4 ± 12.91 147.7 ± 6.21 *** 135.7 ± 4.17 ****	6
	Male	WT Cx43^+/− ^ +^37,43^Gap27	−8.58 ± 0.24 −7.35 ± 0.09 *** −8.94 ± 0.09	176.3 ± 14.98 145.9 ± 7.40 **** 133.6 ± 4.17 ****	6

LogEC_50_ indicates logarithm of median effective concentration. E_max_ represents maximal contraction. Changes in LogEC_50_ and E_max_ between two different groups were analyzed by Student’s unpaired *t*-test. No significant differences between female and male mice were found in either LogEC_50_ or E_max_. *** *p* < 0.001, **** *p* < 0.0001 compared to WT within same experimental group, n = 6 per group. Data are shown as mean ± S.E.M

**Table 2:** The relaxation responses to ACh in IPAs from female and male WT and Cx43^+/−^ mice.

Agonist	Groups		LogEC_50_ (M)	R_max_ (%)	n
ACh	Female	WT Cx43^+/−^ +^37,43^Gap27	−7.58 ± 0.07 −7.54 ± 0.17 −7.51 ± 0.21	69.76 ± 1.97 39.47 ± 2.21 *** 32.70 ± 2.30 ***	6
	Male	WT Cx43^+/−^ +^37,43^Gap27	−8.11 ± 0.07 −7.57 ± 0.14 −7.81 ± 0.16	65.82 ± 1.21 40.79 ± 1.96 **** 27.24 ± 1.13 ****	6

LogEC_50_ indicates logarithm of median effective concentration. R_max_ represents maximal relaxation effect. Changes in LogEC_50_ and R_max_ between two different groups were analyzed by Student’s unpaired *t*-test. No significant differences between female and male were found in either LogEC_50_ or R_max_. *** *p* < 0.001, **** *p* < 0.0001 compared to WT within same experimental group, n = 6 per group. Data are shown as mean ± S.E.M.

**Table 3:** The relaxation responses to SNAP in IPAs from female and male WT and Cx43^+/−^ mice.

Agonist	Groups		LogEC_50_ (M)	R_max_ (%)	n
SNAP	Female	WT Cx43^+/−^ +^37,43^Gap27	−6.88 ± 0.46 −7.19 ± 0.11 −7.46 ± 0.13	103.6 ± 25.11 67.20 ± 3.39 **** 69.74 ± 4.21 ****	6
	Male	WT Cx43^+/−^ +^37,43^Gap27	−6.85 ± 0.32 −7.20 ± 0.11 −7.47 ± 0.14	99.90 ± 16.88 66.34 ± 3.44 **** 69.44 ± 4.24 ****	6

LogEC_50_ indicates logarithm of median effective concentration. R_max_ represents maximal relaxation effect. Changes in LogEC_50_ and R_max_ between two different groups were analyzed by Student’s unpaired *t*-test. No significant differences between female and male were found in either LogEC_50_ or R_max_. **** *p* < 0.0001 compared to WT within same experimental group, n = 6 per group. Data are shown as mean ± S.E.M.

## Data Availability

No new data were created or analyzed in this study.
